# NETs: a new target for autoimmune disease

**DOI:** 10.3389/fimmu.2025.1646527

**Published:** 2025-10-23

**Authors:** Ran Tang, Jiahua Yin, Zhifang Qin, Min Zhang, Xiaoyi Jia

**Affiliations:** ^1^ School of Pharmacy, Anhui University of Chinese Medicine, Hefei, Anhui, China; ^2^ Anhui Province Key Laboratory of Research & Development of Chinese Medicine, Hefei, Anhui, China; ^3^ Department of Rheumatology and Immunology, The First Affiliated Hospital of USTC, Division of Life Sciences and Medicine, University of Science and Technology of China, Hefei, Anhui, China

**Keywords:** neutrophil, neutrophil extracellular traps, autoimmune disease, mechanism, targeted therapy

## Abstract

Neutrophil extracellular traps (NETs) are reticular fiber structures released by neutrophils in response to various stimuli. Although NETs have antibacterial defense functions, their excessive formation has been proven to accelerate the progression of autoimmune diseases. Increasing studies have shown that NETs play an important role in the pathogenesis of autoimmune diseases. The pathogenesis of recent advances in autoimmune disease research, with a focus on the role of NETs in the etiology and pathogenesis of these disorders, and summarizes the current treatment strategies targeting NETs, aiming to provide new directions for the treatment of autoimmune diseases.

## 1 Introduction

Neutrophils are the most abundant immune cells in the human body and constitute the first defense against pathogen invasion, playing a crucial role in host immunity (1). Typically, neutrophils degranulate by releasing antibacterial and proteolytic enzymes, then perform phagocytosis to kill invading microorganisms; however, when encountering large biological structures that cannot be engulfed (e.g., fungi and parasites), they undergo a distinct process to release DNA, histones, and granular proteins—such as neutrophil elastase (NE) and myeloperoxidase (MPO)—thus forming `neutrophil extracellular traps (NETs) (2). These NETs immobilize, kill, and degrade the pathogens extracellularly through the action of associated proteolytic enzymes.

NETs are involved in many autoimmune diseases and are thought to be crucial in the inflammatory process. Although NETs are beneficial during infection, they may play a harmful role in inflammatory, autoimmune, and other pathophysiological conditions (3–5). NETs promote inflammatory processes by releasing active molecules such as hazardous associated molecular patterns (DAMPs), histones, and extracellular active lyases, leading to further immune responses (6). Thus, NETs can also serve as a potential source of autoantigens that bind to associated autoantibodies produced by inflammatory autoimmune diseases. In autoimmune diseases, including gouty arthritis (GA), systemic lupus erythematosus (SLE), rheumatoid arthritis (RA), psoriasis, antineutrophil cytoplasmic antibody (ANCA)-associated vasculitis (AAV), antiphospholipid syndrome (APS), and type 1 diabetes mellitus (T1DM), NETs exhibit aberrant accumulation and impaired clearance. Based on gradient density, neutrophils are classified into low-density neutrophils (LDNs) and normal-density neutrophils subgroups. LDNs is more likely to form NETs in patients with SLE and psoriasis, which may explain the link between the disease and NETs formation (7, 8). In addition, the composition of NETs may vary according to different stimuli, as well as the diseases associated with them (9). In some cases, NETs may also have anti-inflammatory features (10).

Therefore, this article will elucidate the role of NETs in autoimmune diseases from the perspective of their formation and function, and explore their potential as therapeutic targets, thereby providing new insights for the clinical treatment of autoimmune diseases.

## 2 Overview of NETs

### 2.1 Formation of NETs

**Figure 1:**
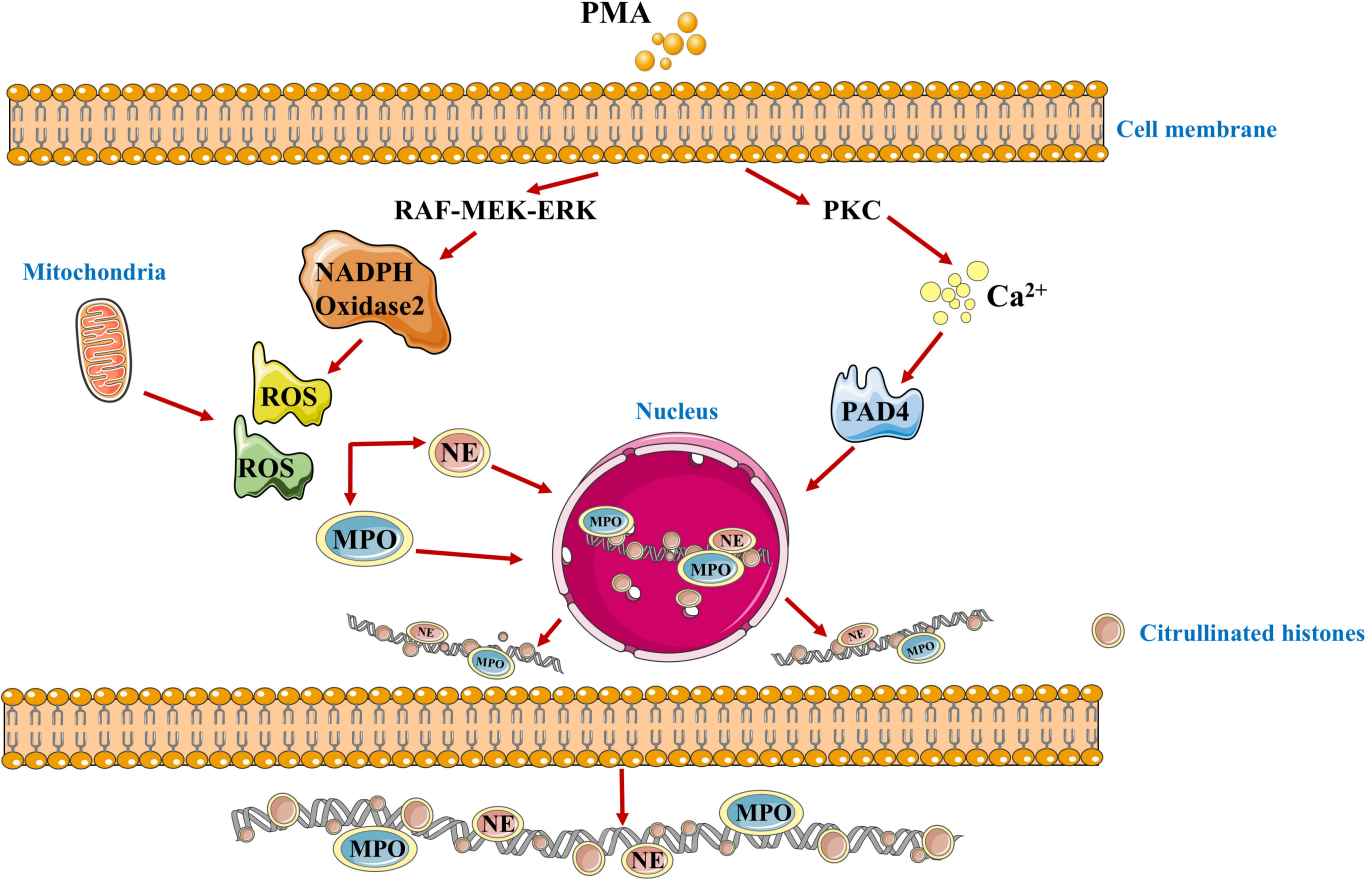
The schematic diagram of the formation mechanism of NETs. PMA, Phorbol-12-myristate-13-acetate; PKC, protein kinase C; ROS, Reactive oxygen species; PAD4, peptidyl arginine xdeiminase 4; MPO, myeloperoxidase; NE, neutrophilic elastase.

The concept of NETs was first proposed by Brinkmann et al. in 2004, who found that neutrophils have a novel mode of death, which is different from cell necrosis and apoptosis (11). Through the breakdown of the neutrophil plasma membrane, a highly active mixture of nucleic acids and proteins is released outside the cell, forming this smooth filamentous structure with an DNA as a skeleton to which various protein particles are anchored called NETs (12). The formation of NETs is triggered by a variety of factors, including cytokines, bacteria, fungi, viruses and protozoa (13, 14). For example, stimulated by Phorbol-12-myristate-13-acetate (PMA), PMA can promote the assembly and activation of nicotinamide adenine dinucleotide phosphate oxidase (NOX) and induce the production of reactive oxygen species (ROS) without forming phagosomes (15, 16). The RAF-MEK-ERK pathway is located upstream of NOX, regulates the production of ROS. However, there are also NOX independent ROS, which are produced by mitochondria (17). ROS damage secretory granules and lysosome membranes, resulting in the release of NE and MPO. NE is first translocated to the nucleus, cutting some specific histones and promoting chromatin depolymerization. Subsequently, MPO also enters the nucleus and collaborates with NE to promote chromatin depolymerization (18). PMA can also activate peptidyl arginine deiminase 4 (PAD4) by binding to protein kinase C (PKC) to induce the release of intracellular calcium ions. PAD4 can dominate histone arginine residues to form citrulline residues, reduce the positive charge, weaken the electrostatic binding force with DNA, and thus depolymerize chromatin (19). It can be seen that the core process in the formation of NETs is chromatin deaggregation, which requires the participation of ROS, NE, MPO and PAD4. Depolymerized chromatin is released from the ruptured nucleus into the cytoplasm, and together with other substances in the cytoplasm such as MPO and NE, it is discharged into the extracellular space through membrane tearing, resulting in the death of neutrophils (20). The mechanism of NETs formation is shown in **Figure 1**.

### 2.2 Functions of NETs

NETs are a double-edged sword. On the one hand, NETs have been shown to play a positive role in controlling bacterial infections (21, 22). Substances such as histone, NE, MPO, cathepsin G, lactoferrin, and antimicrobial peptides can protect wounds and prevent the spread of infection. Histone proteins play an important role in the decomposition of bacterial cell membrane (23). In anti-HIV, influenza, and novel coronavirus, viruses stimulate the formation of NETs via Toll Like Receptor 4 (TLR4), TLR7, and TLR8, NETs inhibit viral replication by capturing the virus or blocking the PKC pathway (24–26). In terms of anti-parasite, immune cells play an irreplaceable role in host defense. For example, neutrophils play a protective role in toxoplasmosis infected fibroblasts (27). Neutrophils can also produce antimicrobial factors to stop the spread of leishmania (28). On the other hand, excessive formation of NETs or inadequate clearance by the body may result in uncontrolled inflammatory response. NETs can regulate congenital and adaptive immune disorders through various mechanisms, and NETs can also amplify inflammatory responses, possibly worsening diseases and even organ damage (29, 30). Because some of the released proteins have non-specific effects, they will directly cause damage to other cells, form immune complexes, induce the production of autoantibodies, and result in pathological tissue damage (31). It has been found that there are a large number of circulating NETs in patients with sepsis, and their presence is associated with poor prognosis and multiple organ failure (32–34). NETs, through their pro-inflammatory and cytotoxic effects, can promote the progression of various diseases including autoimmune disorders, thrombotic conditions, cancer metastasis and progression, as well as severe COVID-19 (35). Histones have DAMPs that increase the release of pro-inflammatory cytokines and activate the Pyrin Domain Containing Protein 3 (NLRP3) inflammasome to further amplify the inflammatory response (36). Induce cytotoxicity and increase the production of ROS, cause endothelial dysfunction and induce organ damage.

Therefore, although NETs constitute a crucial antimicrobial defense mechanism, their uncontrolled release poses a significant threat to host tissues. Thus, the following section will focus on how the detrimental effects of NETs—resulting from either excessive formation or impaired clearance—play a critical role in the pathogenesis of various autoimmune diseases.

## 3 NETs and autoimmune diseases

**Figure 2:**
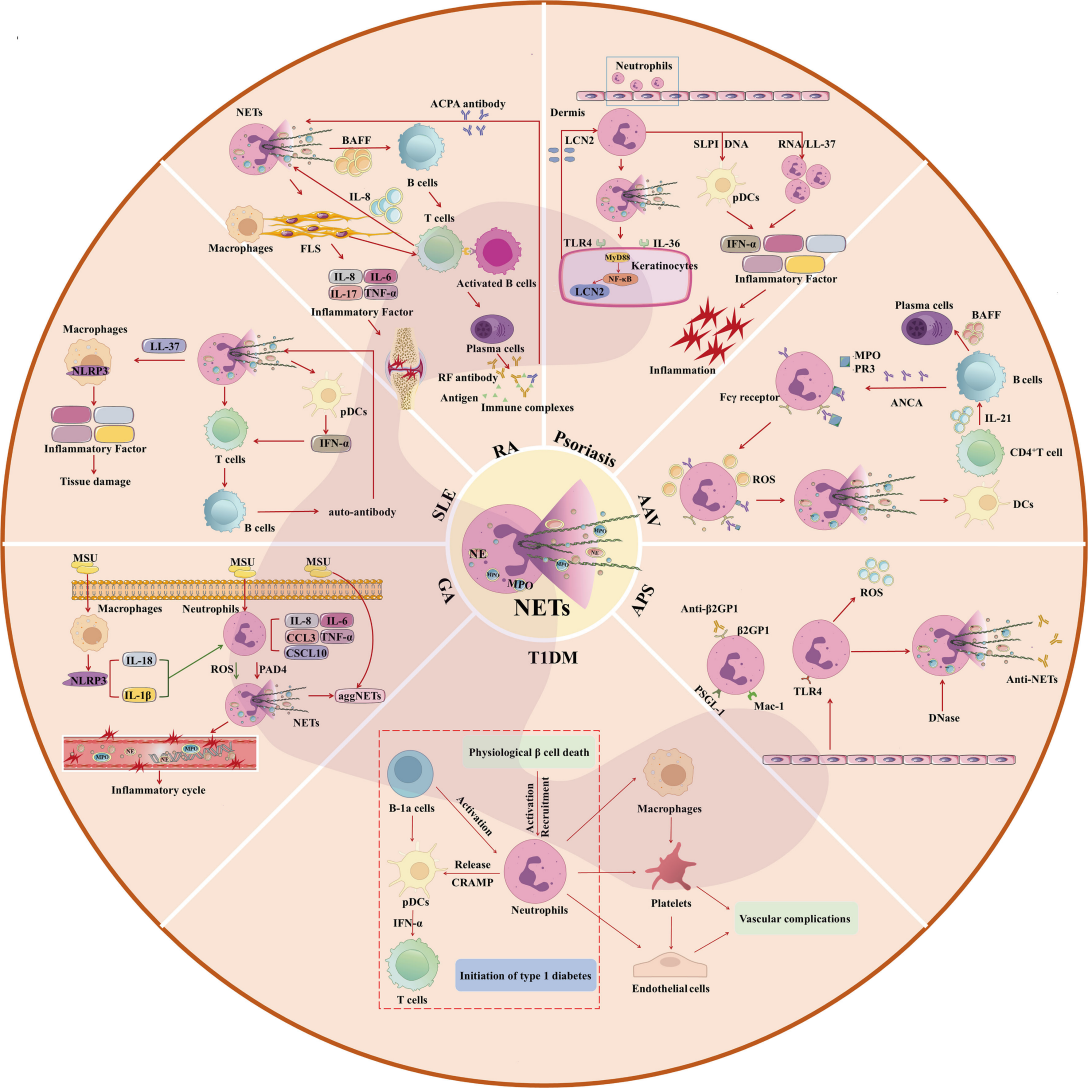
Mechanism of Neutrophils and NETs in Autoimmune Diseases (GA, SLE, RA, Psoriasis, AAV, APS, T1DM). MSU, Monosodium urate; aggNETs, aggregated NETs; NETs, neutrophil extracellular traps. pDCs, Plasmacytoid Dendritic Cells; IFN-α, interferon-α; TNF-α, Tumor Necrosis Factor-α; ACPA, anti-citrullinated protein antibody; RF, rheumatoid factor; BAFF, B Cell Activating Factor; LCN2, Lipocalin-2; MyD88, Myeloid differentiation factor-88; NF-κB, nuclear factor-kappaB; TLR4, Toll-like receptor 4; PR3, protease 3; ANCA, anti-neutrophil cytoplasmic antibody; DCs, Dendritic cells; MPO, myeloperoxidase; ROS, Reactive oxygen species; β2GP1, β2-glycoprotein-1; PSGL-1, P-selectin glycoprotein-1; DNase, deoxyribonuclease; TLR4, Toll-like receptor 4; MAC-1, Macrophage-1; CRAMP, cathelicidin-related antimicrobial peptide.

Autoimmune diseases are caused by the breakdown of the balance between the body’s immune defense and its own antigens, resulting in immune response and damage to its own tissues. At this time, autoimmune cells unable to distinguish between “self” and “non-self” components, the immune system is abnormal, the body produces antibodies to attack itself, causing organ and tissue damage (37). Neutrophils play an irreplaceable role in autoimmune diseases, the excessive formation or insufficient clearance of NETs affects the course of autoimmune disease. Therefore, the study of NETs may be a new direction in the treatment of autoimmune diseases, and possibly the prevention of other diseases associated with the disease. The mechanism of neutrophils and NETs in autoimmune diseases is shown in **Figure 2**.

### 3.1 NETs and GA

GA, the most common inflammatory arthritis, is a multifactorial autoinflammatory disease uniquely characterized by the deposition of monosodium urate (MSU) crystals within joints, which triggers acute and painful synovitis (38, 39). During the onset of acute GA, the accumulation of MSU crystals can induce a large infiltration of inflammatory cells (such as neutrophils and monocytes) into the MSU crystal deposit site of the patient. The release of from these immune cells further triggers the release of various pro-inflammatory cytokines and chemokines, which upregulates selectin and integrin on the surface of endothelial cell lumen, and further enhances neutrophil recruitment (40). Upon contact with MSU crystals, neutrophils release a range of inflammatory mediators—including Tumor Necrosis Factor alpha (TNF-α), Interleukin-6 (IL-6), as well as neutrophil inducers (e.g., IL-8) and activators (such as CCL3 and CXCL10) (41). Furthermore, MSU crystals activate infiltrating neutrophils not only to secrete cytokines but also to form NETs through two principal mechanisms: a ROS-independent vital pathway involving calcium-mediated direct activation of PAD4, and a ROS-dependent suicidal pathway driven by calcium/NOX-generated ROS leading to PAD4 activation—both of which ultimately result in NETs release (42). Macrophage phagocytosis of MSU crystals activates the NLRP3 inflammasome, prompting release of IL-1β and IL-18, which recruit neutrophils that undergo oxidative burst (a rapid ROS-producing process) and form NETs through genomic DNA and granular protein release (43). These NETs components—notably histones and DNA—directly damage tissues such as vascular endothelium, inducing further release of inflammatory mediators like Adenosine Triphosphate and uric acid and thereby sustaining a feed-forward inflammatory cycle (44). NETs release proinflammatory mediators in the early stages of neutrophil recruitment or when the number of neutrophils is comparable to that of peripheral blood (40). Conversely, a high density of neutrophils—such as that found in the synovial fluid of GA or within highly infiltrated inflammatory tissues—promotes the formation of aggregated NETs (aggNETs) both *in vivo* and *in vitro* (45). These aggNETs can be covered on the surface of MSU crystals to isolate them from inflammatory mediators, thus promoting the formation of gout stones and indirectly alleviating the damage caused by MSU crystals to the body (46). Thus, in GA, the formation of NETs has a double-edged sword effect: on the one hand, NETs can package and isolate MSU crystals or adhere to apoptotic cells and clear them, inhibit inflammatory response and protect the body by controlling the production of cytokines by proteases, which shows that NETs play a role in promoting and alleviating inflammation. On the other hand, NETs release various damage-related molecular modes (histone or DNA-activated TLR or NLRP3 inflammatory bodies), which aggravates the inflammatory response by releasing pro-inflammatory factors or direct action, and excessive NETs will form aggNETs, promote the formation of gout, cause bone erosion, and induce chronic inflammatory reaction. Given the unique role of NETs in GA, targeting NETs is considered a highly attractive and promising therapeutic approach for treating GA.

### 3.2 NETs and SLE

SLE is a complex multi-system autoimmune disease involving epigenetic, genetic, ecological, and environmental factors (47). It is characterized by the presence of autoantibodies targeting nuclear and cytoplasmic antigens, with antinuclear antibody serving as a key serological marker (48). Patients with SLE produce a variety of autoantibodies, among which anti-dsDNA antibodies are highly specific and contribute to disease pathogenesis (49). NETs play a crucial role in SLE through multiple mechanisms. Anti-dsDNA antibodies can be components of NETs, and impaired NETs clearance—due to deoxyribonuclease (DNase) inhibition, DNase inhibitors, or anti-NET antibodies—leads to NETs accumulation, further elevating anti-dsDNA antibody titers and activating the complement system, thereby perpetuating a vicious cycle of inflammation (50).

Studies have indicated that levels of NETs are elevated in patients with SLE. NET-associated proteins, such as LL-37, can promote hyperactivation of the NLRP3 inflammasome in adjacent macrophages, leading to the release of large quantities of inflammatory cytokines and thereby causing severe tissue damage (51). In this process, released IL-18 further stimulates neutrophils to produce more NETs (52). Moreover, inflammasome-mediated activation of gasdermin D is also a key factor in NET formation (53, 54). Additionally, NETs can lower the activation threshold of T cells and promote T cell activation via T cell receptor signaling, thereby linking innate and adaptive immune responses (55). NETs in SLE are enriched in ox-mtDNA and citrullinated histones, which act as potent autoantigens. These structures activate Toll-like receptors and intracellular nucleic acid sensors, triggering Interferon Alpha (IFN-α) production by plasmacytoid dendritic cells (pDCs). IFN-α promotes dendritic cell maturation, T-cell activation, and autoantibody production by B cells, further stimulating NETs release (56, 57).

LDNs are a distinct subpopulation of neutrophils in patients with SLE (58). LDNs is significantly increased in the peripheral blood of SLE patients. LDNs from SLE patients demonstrate the capacity to activate T cells and induce the release of cytokines, including IFN-γ and TNF-α, a function not exhibited by their normal-density neutrophil counterparts (59). CD10^+^ LDNs exhibit a mature polymorphonuclear morphology, express high levels of type I interferon-stimulated genes (ISG15, MX1) and proinflammatory cytokines (IL-6, IL-8, TNF-α), and demonstrate enhanced NET-forming capacity (60). These NETs contain ox-mtDNA and citrullinated histone H4, which not only act as autoantigens driving anti-dsDNA antibody production via B-cell TLR9 activation but also stimulate the NLRP3 inflammasome in macrophages, promoting pyroptosis and IL-1β/IL-18 release (51). This cascade further potentiates Neutrophil Extracellular Traposis (NETosis), while IFN-α released from pDCs feedback enhances NETs formation and impairs endothelial repair, establishing a self-sustaining inflammatory loop.

Given the core role of NETs in the pathogenesis of SLE and their detectability in patient serum and tissues, NETs are expected to serve as biomarkers for disease activity, organ involvement, and treatment response.

### 3.3 NETs and RA

RA is a systemic inflammatory autoimmune disease characterized by joint inflammation and bone damage (61). It is characterized by persistent synovitis, systemic inflammation, the presence of autoantibodies, and the production of a large number of inflammatory cytokines, which can lead to articular cartilage and bone damage (62). The serological hallmark of RA is anti-citrullinated protein antibodies (ACPA) (63). Citrullinated histones are thought to be a persistent source of B cell antigens that promote the production of new ACPA (64).

It was found that the synovial fluid in RA patients was infiltrated by neutrophils, which were prone to form NETs (65, 66). When neutrophils are activated, a large number of histones are citrullinated by PAD4, which is a key step in chromatin decondensation and NETs release (67, 68). About 70% of the proteins in NETs are histones (69). Citrullinated antigens on NETs play a critical role in initiating and perpetuating autoimmunity and ACPA production (70). Therefore, ACPA-related immune responses and the formation of NETs play an important role in the pathogenesis of RA. Studies have shown that circulating neutrophils in patients with RA are more likely than those in healthy subjects to undergo NETosis (71, 72). As in other autoimmune diseases, NETs act as a source of extracellular autoantigens, leading to excessive innate and adaptive immune responses within the joint and subsequent tissue damage.

NETs promote synovial inflammation by stimulating the release of pro-inflammatory cytokines such as IL-6, IL-8, TNF-α, and IL-17 from macrophages and fibroblast-like synoviocytes (FLSs) (73). They also enhance cartilage damage through the internalization of arthritogenic peptides by FLSs via the RAGE-TLR9 pathway, upregulation of MHC class II, and activation of T cells and B cells, leading to ACPA production and inflammatory spread. Additionally, NET-derived enzymes such as NE, matrix metalloproteinase-8 (MMP8), and MMP9 contribute to cartilage matrix degradation (65, 74). Activated neutrophils release B-cell Activating Factor (BAFF) and activate B cells (75). Then, the activated B cells release cytokines to cascade with other immune cells, and B-cell-derived IL-8 recruits neutrophils to the synovial membrane (76). Additionally, B cells, with the help of T cells, promote the production of auto-antibodies. Some of these plasma cells produce a large number of auto-antibodies, including RF and ACPA; these formed immune complexes activate the complement pathway and promote inflammation, which is particularly abundant in RA (77, 78).

Studies have confirmed that the release of NETs exacerbates the occurrence and development of RA (79). ACPA, rheumatoid factor, and inflammatory cytokines (TNF-α, IL-17) can enhance the formation of NETs. In the pathogenesis of RA, NETs repeatedly stimulate the body to produce autoimmune responses through exposure to autoantigens, which aggravates NETosis, forming a vicious cycle and leading to a sustained inflammatory response. *In vitro* and *in vivo* experiments have shown that during the pathogenesis of RA, neutrophils undergo significant activation and death, inducing the formation of NETs and thus exacerbating their own apoptosis (80). Therefore, inhibiting the formation of NETs may provide a new direction for the treatment of RA.

### 3.4 NETs and Psoriasis

Psoriasis is a chronic inflammatory systemic disease with a genetic basis, characterized by symmetrical erythematous skin lesions covered with silvery-white scales (81, 82). While its exact cause remains unknown, neutrophils are among the earliest cells infiltrating nascent psoriatic plaques, and their epidermal accumulation is a hallmark of the disease (83).

Recent evidence highlights the significant role of NETs in psoriasis pathogenesis. Stimulating keratinocytes to produce high levels of various inflammatory mediators induces TLR4 expression (84). The endogenous neutrophil-derived TLR4 ligand then acts synergistically with IL-36 to induce the production of LCN2 through MyD88 and NF-kB activation signaling (85). In turn, upregulated LCN2 regulates the formation of NETs and neutrophil migration, enhancing and maintaining the inflammatory response (86). Circulating neutrophils in psoriasis patients exhibit a heightened tendency for spontaneous or stimulus-induced NETosis, which correlates with disease severity (87). These neutrophils, including an increased population of LDNs, are primed for NETs release (88). Notably, exosomes derived from keratinocytes treated with psoriasis-related cytokines (e.g., IL-17A, IL-22, IFN-γ, TNF-α) can activate normal neutrophils, leading to NETs formation via NF-κB and p38 mitogen-activated protein kinase (MAPK) signaling pathways (89).

Within psoriatic lesions, NETotic neutrophils are found in both the epidermis (e.g., within Munro’s microabscesses) and dermis. These neutrophils can produce IL-17 through NETs formation, contributing to further neutrophil recruitment and sustained inflammation (90). NETs also facilitate Th17 cell differentiation and induce the expression of antimicrobial peptides like human β-defensin-2 in keratinocytes (87, 91). Moreover, NET-derived components form complexes such as DNA/cathepsin G/secretory leukocyte protease inhibitor and RNA/LL-37, which activate pDCs and neighboring neutrophils, respectively (92, 93). These interactions promote the production of type I interferons and proinflammatory cytokines, amplifying the inflammatory cascade.Interestingly, dimethylfumarate—a therapeutic agent for psoriasis—inhibits neutrophil activation, including NETs formation, suggesting that targeting NETosis may be a viable treatment strategy.

### 3.5 NETs and AAV

AAV is a systemic necrotizing small vasculitis that includes Wegener’s granulomatosis, eosinophilic granuloma with vasculitis, and microvasculitis (94). ANCA is a specific antibody that targets MPO and Recombinant Proteinase 3 (PR3) (95). The study suggests that ANCA may be involved in the activation of NETs formation in patients with AAV. AAV occurs when ANCA binds to autoantigens PR3 and MPO, which are granular proteins found on the surface of neutrophils that are associated with GPA and MPA, respectively (96). NETs also contain the targeted antigen MPO (stored within neutrophilic granulocyte) or PR3 (expressed on the membrane of resting neutrophils) whose expression rises when neutrophils are activated by cytokines (97). Studies have shown that NETs is also modified by MPO and PR3 *in vitro* and *in vivo* immunofluorescence in AAV necrotic lesions (98). For example, the co-localization of DNA, MPO, and PR3 in the kidney tissue of patients with small vasculitis (SVV) glomerulonephritis indicates the presence of NETs and ANCA antigens in the inflammatory tissue (99). In patients with low NETs degradation activity, these NETs persist, particularly MPO and PR3. These antigens are presented to CD4^+^T cells via dendritic cells, producing ANCA (100). Neutrophils express MPO and PR3 on the plasma membrane, and PR3-ancas and MPO-ANCAs bind to them. At the same time, these crystallizable fragment (Fc) regions of ANCA bind to the Fc-γ receptor on neutrophils (101). This binding induces hyperactivation of neutrophils, resulting in abnormal cytokine production, while releasing ROS and lyases, which further form NETs and damage vascular endothelial cells. In addition to ANCA, BAFF are produced by activated neutrophils, and CD4^+^T cells (via IL-21) stimulate B cells, enabling continuous ANCA production (102). The study found that higher levels of MPO-DNA were detected in the serum of patients with active AAV compared to those with AAV in remission (103). However, another study did not find a difference in serum MPO-DNA levels between patients with active AAV and those with AAV in remission (104). This discrepancy may be related to differences in NETs clearance capacity among individual patients, such as reduced serum DNase I activity or impaired macrophage phagocytic function, which can lead to NETs accumulation that does not fully correlate with current clinical activity (105, 106). These findings indicate that while NETs are clearly present in patients with AAV, their utility as biomarkers for assessing disease activity remains to be determined. Furthermore, despite the potential of NET-associated biomarkers (e.g., MPO-DNA, citrullinated histones, and cell-free DNA) to aid in diagnosis, prognostic evaluation, and relapse prediction, further standardization and validation are still required.

NETs contribute to the progression of AAV through multiple mechanisms. On one hand, NETs are not only involved in the initiation of ANCA autoimmune responses but also directly cause vascular damage via histone-mediated cytotoxicity (107). On the other hand, the persistence of NETs is closely associated with an imbalance in their clearance. Studies have demonstrated that the endogenous degrading factor DNase1 can effectively degrade NETs, while intravenous immunoglobulin (IVIG) exhibits therapeutic potential by significantly inhibiting NET formation (108, 109). In therapeutic approaches, targeted strategies addressing NETs formation and clearance have emerged as research hotspots, including: C5a receptor antagonists (e.g., Avacopan), Syk inhibitors, PAD4 inhibitors, and recombinant DNase I. To sum up, NETs play an extremely important role in the pathogenesis of AAV. NETs can be used as the information of disease diagnosis and the target of future treatment. Effective intervention in the formation of NETs is expected to provide new ideas for the treatment of autoimmune vasculitis.

### 3.6 NETs and APS

APS is an autoimmune disorder associated with elevated levels of antiphospholipid antibodies (aPL), characterized by arterial, venous, or small vessel thrombosis or recurrent early pregnancy loss, fetal loss (110). aPL is a general term for antibodies to phospholipids and surface proteins, including lupus anticoagulants, anti-β2-glycoprotein-1, and anti-cardiolipin (111, 112). aPL is known to promote thrombosis by activating endothelial cells, monocytes, and platelets. Several mechanisms contribute to the release of NETs in APS. Anti-β2GP1 antibodies recognize β2GP1 bound to the surface of neutrophils, leading to the upregulation of adhesion molecules PSGL-1 and Mac-1 (113). This enhances their adherence to the endothelium. Subsequently, under the influence of this endothelial adhesion, TLR4 signaling, and potential interferon stimulation, APS neutrophils become activated and release ROS and NETs (114). Furthermore, anti-NETs antibodies present in APS may impair the clearance of NETs by inhibiting circulating DNase, preventing their effective degradation (115, 116). Studies have found that NETs play a key role in the involvement of platelets and neutrophils in the formation, stabilization, and growth of peripheral and coronary thrombosis (117). In patients with APS, increased NETs release is associated with autoimmunity and inflammation, driven by stimuli such as immune complexes, autoantibodies and complement activation (118). Seminal work by Yalavarthi et al. showed that IgG from APS patients stimulates NETosis in control neutrophils via mechanisms dependent on ROS and TLR4 signaling (119, 120). Moreover, impaired degradation of NETs—due to DNase inhibitors or anti-NET antibodies—further contributes to NETs persistence and thrombotic risk (121). Inhibition of NETs release may have potential benefits in patients with APS.

Experimental studies have demonstrated that serum and purified IgG isolated from patients with APS can induce neutrophils to release NETs (122). Furthermore, inhibition of ROS production or blockade of TLR4 signaling has been shown to reduce NET formation (123). In an animal model of APS, administration of patient-derived IgG was associated with increased thrombosis; conversely, degradation of NETs via DNase I treatment or depletion of neutrophils significantly attenuated thrombotic events (124). These findings suggest that modulating NETs formation or enhancing their clearance may represent a promising therapeutic strategy for APS.

### 3.7 NETs and T1D

T1DM is an autoimmune disease characterized by destruction of islet β cells, characterized by elevated blood glucose levels, often accompanied by absolute lack of endogenous insulin (125). Although the pathogenesis of T1DM is unknown, physiologic β cell death is a predisposing factor in the development of the disease through recruitment and activation of neutrophils, which penetrate the pancreas. In the pancreas, neutrophils can release CRAMP, pDCs can be induced to produce interferons alpha (126). The interaction between immune cells is necessary to induce a diabetic T cell response that subsequently leads to the development of T1DM (127). In addition, interactions between neutrophils and other non-immune cells, such as platelets in the blood or vascular endothelial cells, are thought to play an important role in the microvascular and macrovascular complications of diabetes. The number of circulating neutrophils decreased in T1DM patients and high-risk subjects before symptoms. Previous studies have shown that neutrophils infiltrate the pancreas before onset and form NETs within it, exhibiting strong pro-inflammatory biological activity (128). NETs is an important link for neutrophils to participate in the occurrence and development of T1DM, T1DM neutrophils express high levels of PAD4 and produce more NETs. In human T1DM, reduced circulating neutrophils and elevated NETs markers (e.g., NE, PR3, CitH3) correlate with autoantibody levels and beta cell loss (129). NETs and histones directly damage human islets *in vitro*, an effect reversible with polyanions like Mcbs (130).In the serum of children 10 days after T1DM onset, the levels of NETs, mtDNA and nuclear DNA in peripheral blood were higher than those in healthy children, and T1DM serum could induce normal neutrophils to form NETs (131). However, some studies have found that the levels of NE and PR3 in T1DM subjects decreased significantly, especially in the subjects within three years after diagnosis (132). The levels of NE and PR3 were correlated with the absolute neutrophil count. This may reflect disease stage-dependent changes in neutrophil activity. In the study of T1DM, non-obese diabetic (NOD) mice can spontaneously be T1DM, which is often used to study the pathogenesis and intervention of T1DM (133). You et al. found that the formation of PAD4 dependent NETs is involved in the aggravation of intestinal barrier dysfunction, the production of autoantibodies and the activation of intestinal autoimmune T cells in DSS-induced colitis and PAD4 knockout experiments in NOD mice, and then these cells migrated to pancreatic lymph nodes to cause injury (134). Neutrophils also promote early autoimmunity in NOD mice via pDC activation and IFN production (135). In female NOD mice, physiological β cell death induced the recruitment and activation of B-1a cells, neutrophils and plasma cell-like dendritic cells (pDC) to the pancreas (136). Activated Bmur1a cells secrete double-stranded DNA-specific IgG and activate neutrophils to form NETs. This DNA-specific IgG activates pDCs through Toll-like receptors, resulting in the production of IFN-α in islets and the formation of T1DM (137). Notably, PAD4 inhibition prevents diabetes in NOD mice, underscoring the role of NETosis (138). NETs impair wound healing and are more pronounced in T1DM, and inhibiting NETs may improve wound healing in diabetes and reduce NETs-driven chronic inflammation (139).

In T1DM, although the level of NETs is uncertain, the presence of NETs directly or indirectly activates innate and adaptive immune responses in the pancreas, damages islet β cells, and participates in the occurrence and development of T1DM. Accumulating evidence from humans and NOD models indicates NETs contribute to islet autoimmunity through cytotoxicity and immune activation. Therefore, the study of NETs may be one of the directions in the treatment of T1DM.

## 4 Drug intervention in NETs to treat autoimmune diseases

A variety of drugs have been used to treat autoimmune diseases in clinic, and their mechanism of action has been gradually explored. Studies have found that a variety of drugs may act on NETs to play a therapeutic.

### 4.1 NE and MPO inhibitors

NE and MPO are key synergistic molecules in the process of NETs formation, as well as core functional components of NETs structure. They collectively participate in immune defense and mediate the amplification of inflammation and tissue damage during the pathogenesis of diseases. Therefore, inhibitors targeting NE and MPO have emerged as potential therapeutic strategies, some of which have advanced into clinical research.

Among NE inhibitors, Sivelestat is a selective, reversible, and competitive small-molecule inhibitor that suppresses NE enzymatic activity by binding to its active site, thereby reducing NETs formation and mitigating inflammatory responses and tissue injury (140). Studies have shown that early administration of Sivelestat in diabetic mouse models significantly reduces the incidence of spontaneous insulitis and autoimmune diabetes (141). Furthermore, this compound has demonstrated therapeutic potential in various animal models of acute respiratory distress syndrome, sepsis, non-alcoholic steatohepatitis, and acute lung injury. Other NE inhibitors that have entered clinical stages include POL6014, PHP-303, Elafin, CHF6333, and alvelestat, all of which inhibit NE activity through a similar competitive mechanism (142–145). On the other hand, MPO inhibitors such as PF-1355 can significantly reduce MPO activity in mouse plasma, thereby inhibiting neutrophil recruitment and vascular edema, and have been used in basic research on immune complex-mediated vasculitis (146). In addition, ceruloplasmin has been shown to decrease plasma MPO activity in mice and inhibit the production of MPO-derived oxidants during inflammation, demonstrating protective effects (147). Recent studies have also indicated that ABAH reduces MPO-dependent hepatocyte death in a non-alcoholic steatohepatitis model, decreases MPO activity in a mouse model of acute stroke, and inhibits MPO activity in sputum from pulmonary cystic fibrosis (148–150). Similarly, compounds such as INV-315, PF-0628999, and AZM198 alleviate inflammatory responses by inhibiting MPO activity (151, 152).

In summary, NE and MPO inhibitors exhibit promising therapeutic effects in various disease models by regulating NETs formation and neutrophil-mediated inflammatory responses. Some compounds have progressed to clinical research stages, offering new directions for the treatment of related inflammatory and autoimmune diseases.

### 4.2 DNase I

DNA serves as the primary structural framework of NETs. DNase I is an enzyme capable of degrading DNA, effectively breaking down the DNA component within NETs, thereby reducing NETs formation (153). Although the early use of recombinant human DNase I (rhDNase I) in the treatment of SLE demonstrated a favourable safety profile, its clinical efficacy was limited; nevertheless, it has been approved for the treatment of cystic fibrosis (CF) (154). Recently, a novel bioenzyme with dual DNase1/DNase1L3 activity has shown significant effects in murine lupus models, effectively suppressing autoantibody production and resisting neutralization by autoantibodies in SLE patients (155). In RA patients, DNase I can also inhibit neutrophil NET generation and mitigate NET-induced thrombosis and endothelial damage (79). On the other hand, advances in production technology have provided crucial support for the clinical application of DNase I. Recent studies indicate that the use of a Pichia pastoris expression system enables successful recombinant production of active human DNase I (156). This breakthrough is expected to substantially reduce manufacturing costs and lay the foundation for large-scale applications in various NET-related diseases.

### 4.3 Targeted IFN preparations

Therapeutic targeting of IFN signaling can reduce NET-induced inflammation and autoimmune responses. For instance, both the JAK inhibitor tofacitinib and the type I IFN inhibitor anifrolumab have been demonstrated in clinical studies to lower NETs levels in SLE patients and improve their clinical symptoms (157, 158).

Current biologic agents for SLE treatment primarily consist of monoclonal antibodies that directly target either IFN-α or the type I IFN receptor (IFNAR). Sifalimumab and rontalizumab are two anti-IFN-α monoclonal antibodies. Among them, sifalimumab has been shown to significantly reduce SLE disease activity, whereas rontalizumab did not demonstrate notable efficacy—the underlying mechanisms remain unclear (159, 160). Anifrolumab, an anti-IFNAR monoclonal antibody, has been approved by the U.S. FDA and the European Union for the treatment of moderate to severe SLE (161). Additionally, QX006N is another monoclonal antibody targeting IFNAR1. It specifically binds to the SD3 domain of IFNAR1, creating steric hindrance that prevents the binding of type I IFN ligands and inhibits the assembly of the IFN/IFNAR1/IFNAR2 complex (162). This agent is currently under investigation for SLE therapy.

### 4.4 PAD4 inhibitor

PAD4 serves as a nuclear promoter that mediates citrullination of histone H3 in neutrophils, facilitating chromatin decondensation and promoting NET formation. Studies have shown that inhibiting PAD4 to suppress NETosis confers protective effects in mouse models of lupus, diabetes, and atherosclerosis without significant adverse events. Cl-amidine inhibits the citrullination of histone H3 by irreversibly binding to PAD4, thereby restraining NET formation (163).Research indicates that Cl-amidine alleviates endothelial dysfunction in SLE mice and reduces the deposition of immune complexes in renal tissues (164). Furthermore, Cl-amidine suppresses the production of NETs and inflammatory cytokines by reducing PAD4 levels in the joint tissues of arthritic mice, thereby ameliorating joint edema (165). Meanwhile, *in vivo* studies demonstrate that GSK484, a reversible PAD4 inhibitor, also inhibits NET release and exerts immunomodulatory effects. It enhances radiosensitivity in colorectal cancer by promoting DNA double-strand breaks and suppresses NET formation both *in vivo* and *in vitro* (166). Administration of GSK484 in CIA mice reduces the expression of synovial MPO, NE, and PAD4, decreases NET generation, attenuates arthritis severity, and inhibits macrophage infiltration, supporting its therapeutic potential (167). In various lupus models, PAD inhibitors can reduce NETs formation while protecting the vasculature, kidneys, and skin from damage. The selective PAD4 inhibitor JBI-589 blocks NET formation and PAD4-dependent citrullination; oral administration in mouse models reduces the incidence and severity of arthritis and inhibits ACPA production (168).

### 4.5 ROS remover

ROS are essential for the formation of NETs. A range of ROS scavengers have demonstrated therapeutic potential in autoimmune diseases. As a scavenger of ROS, N-acetylcysteine (NAC) has been observed to reduce NET generation upon treatment (169). In two clinical studies, NAC administration improved disease outcomes in SLE patients, though related mechanistic investigations remain at an early stage (170). Moreover, MitoTempo, a specific scavenger of mitochondrial ROS, prevented spontaneous NETosis and reduced disease severity in a lupus mouse model (171). Ethyl pyruvate attenuates NET formation and sepsis-induced intestinal injury by inhibiting ROS-mediated activation of MAPK/ERK1/2 and p38 MAPK (172). Additionally, other agents targeting ROS also exhibit efficacy. For instance, diphenyleneiodonium demonstrates significant anti-tumor activity in MYCN-amplified neuroblastoma by targeting MYCN-induced mitochondrial alterations and ROS production, thereby inducing apoptosis and suppressing tumor growth (173).

### 4.6 Traditional Chinese medicine compounds

Traditional Chinese medicine (TCM) represents a precious treasure endowed by nature and is increasingly demonstrating its therapeutic value. Numerous compounds derived from TCM that are currently under preclinical investigation have shown potential for targeting NETs. For instance, triptolide has been found to inhibit NET generation *in vitro* independently of cellular ROS levels (174). In a murine model of RA, it suppressed neutrophil autophagy, NET formation, tissue damage, and inflammation (175). Similarly, tetrandrine exhibited therapeutic effects in an RA model by modulating neutrophil-associated inflammatory responses and inhibiting NET formation (176). Quercetin was shown to reduce neutrophil infiltration, plasma cytokine levels, and autophagy-dependent NET formation (177). Andrographolide decreased joint levels of CXCL2, MPO, and NE, while also reducing neutrophil infiltration in ankle tissues (178). The classical TCM formula Simiao Yong’an Tang inhibited neutrophil migration, promoted apoptosis, and reduced ROS production and NET formation *in vitro* (179). Additionally, emodin alleviated arthritis in AA mice by diminishing neutrophil infiltration, inhibiting the release of pro-inflammatory cytokines (IL-6, IFN-γ, and TNF-α), suppressing autophagy-mediated NETosis, and promoting neutrophil apoptosis (180). Furthermore, our recent study demonstrated that Ermiao San and its primary active components (phellodendrine and atractylenolide-I) exert therapeutic effects against RA by suppressing PAD4 to reduce the formation of NETs (181).

## 5 Conclusion

The burgeoning field of NETosis has fundamentally redefined our understanding of autoimmune pathogenesis, establishing NETs not merely as inflammatory effectors but as central orchestrators that bridge innate and adaptive immunity. NETs contribute to autoimmunity through multiple mechanisms: they serve as a source of autoantigens, amplify inflammatory cascades, activate innate and adaptive immune pathways via Toll-like receptors, inflammasomes, and type I interferon responses, and directly cause tissue damage through cytotoxic components. Their involvement across various autoimmune diseases, including SLE, RA, APS, and T1DM, highlights a shared pathological mechanism rooted in dysregulated NET formation and clearance. Therapeutic strategies targeting NETs, such as inhibitors (e.g., PAD4 inhibitors, neutrophil elastase inhibitors, myeloperoxidase inhibitors, reactive oxygen species (ROS) inhibitors), DNase-based interventions, and biologics targeting interferon signaling pathways, have demonstrated significant potential in both preclinical and clinical studies. Additionally, multi-omics-driven biomarker discovery and exploration of the microbiome–NET axis hold promise for improving diagnosis, subtyping, and personalized treatment. The integration of advanced technologies—such as single-cell analysis, real-time NET imaging, and neutrophil engineering—will be crucial to translate these mechanistic insights into precise clinical interventions, ultimately revolutionizing the management of autoimmune diseases.

## Glossary

AAV: antineutrophil cytoplasmic antibody -associated vasculitis
LL-37: Cathelicidin Antimicrobial Peptide
ACPA: anti-citrullinated protein antibodies
MAPK: mitogen-activated protein kinase
aggNETs: aggregated NETs
MMP8: matrix metalloproteinase-8
ANCA: antineutrophil cytoplasmic antibody
MMP9: matrix metalloproteinase-9
aPL: antiphospholipid antibodies
MPO: myeloperoxidase
aPL: antiphospholipid antibodies
MSU: monosodium urate
APS: antiphospholipid syndrome
NE: neutrophil elastase
BAFF: B-cell Activating Factor
NETosis: Neutrophil Extracellular Traposis
CCL3: C-C chemokine ligand 3
NETs: Neutrophil extracellular traps
CF: cystic fibrosis
NF-κB: Nuclear factor kappa-light-chain-enhancer of activated B cells
CRAMP: Dopamine-Cathelicidin-related antimicrobial peptide
NLRP3: the Pyrin Domain Containing Protein 3
CXCL10: C-X-C motif chemokine ligand 10
NOD: non-obese diabetic
DAMPs: hazardous associated molecular patterns
NOX: nicotinamide adenine dinucleotide phosphate oxidase
DNase: deoxyribonuclease
PAD4: peptidyl arginine deiminase 4
FLSs: fibroblast-like synoviocytes
pDCs: plasmacytoid dendritic cells
GA: gouty arthritis
PKC: protein kinase C
IFN-α: Interferon Alpha
PMA: Phorbol-12-myristate-13-acetate
IFN-γ: Interferon-gamma
PR3: Proteinase 3
IFN-γ: Interferon-gamma
PR3: Recombinant Proteinase 3
IL-17: Interleukin-17
RA: rheumatoid arthritis
IL-17A: Interleukin-17A
rhDNase I: recombinant human DNase I
IL-18: Interleukin-18
ROS: reactive oxygen species
IL-1β: Interleukin-1β
SLE: systemic lupus erythematosus
IL-21: Interleukin-21
T1DM: type 1 diabetes mellitus
IL-22: Interleukin-22
TLR4: Toll Like Receptor 4
IL-6: Interleukin-6
TLR7: Toll Like Receptor 7
IL-8: Interleukin-8
TLR8: Toll Like Receptor 8
IVIG: Intravenous immunoglobulin
TLR9: Toll Like Receptor 9
LDNs: low-density neutrophils
TNF-α: Tumor Necrosis Factor alpha
